# Supplementary material for: Modelling Skylarks (Alauda arvensis) to Predict Impacts of Changes in Land Management and Policy: Development and Testing of an Agent-Based Model
Source: PLoS One. 2013 Jun 6;8(6):e65803. doi: 10.1371/journal.pone.0065803 (PMC3675089; doi:10.1371/journal.pone.0065803)
Supplement: Supporting Information S4 — The skylark ODdox as a zipped archive. (ZIP) [file pone.0065803.s004.zip › Skylark_ODdox/class_t_animal.html]

ALMaSS Skylark ODdox: TAnimal Class Reference


ALMaSS Skylark ODdox
 2.0


- Main Page
- Related Pages
- Classes
- Files

- Class List
- Class Index
- Class Hierarchy
- Class Members

Public Member Functions |
Protected Member Functions |
Protected Attributes

TAnimal Class Reference

The base class for all ALMaSS animal classes.
More...

`#include <populationmanager.h>`

List of all members.

|  |  |
| --- | --- |
| Public Member Functions | |
| virtual void | BeginStep (void) |
| void | CheckManagement (void) |
| void | CheckManagementXY (int x, int y) |
| virtual void | CopyMyself () |
| virtual void | Dying () |
| virtual void | EndStep (void) |
| virtual void | KillThis () |
| virtual bool | OnFarmEvent (FarmToDo) |
| void | SetX (int a\_x) |
| void | SetY (int a\_y) |
| virtual void | Step (void) |
| int | Supply\_m\_Location\_x () |
| int | Supply\_m\_Location\_y () |
| unsigned | SupplyFarmOwnerRef () |
| int | SupplyPolygonRef () |
| AnimalPosition | SupplyPosition () |
|  | TAnimal (int x, int y, Landscape \*L) |
| virtual int | WhatState () |
| Public Member Functions inherited from TALMaSSObject | |
| void | OnArrayBoundsError () |
|  | TALMaSSObject () |
| virtual | ~TALMaSSObject () |

|  |  |
| --- | --- |
| Protected Member Functions | |
| void | CorrectWrapRound (void) |
|  | Corrects wrap around co-ordinate problems. |

|  |  |
| --- | --- |
| Protected Attributes | |
| int | m\_Location\_x |
| int | m\_Location\_y |
| Landscape \* | m\_OurLandscape |

|  |  |
| --- | --- |
| Additional Inherited Members | |
| Public Attributes inherited from TALMaSSObject | |
| int | CurrentStateNo |
| bool | StepDone |

---

## Detailed Description

The base class for all ALMaSS animal classes.

Includes all the functionality required to be handled by classes derived from Population\_Manager, hence a number of empty methods that MUST be reimplemented in descendent classes e.g. CopyMyself()

---

## Constructor & Destructor Documentation

|  |  |  |  |
| --- | --- | --- | --- |
| TAnimal::TAnimal | ( | int | *x*, |
|  |  | int | *y*, |
|  |  | Landscape \* | *L* |
|  | ) |  |  |

TAnimal Constructor

{

m\_OurLandscape = L;

m\_Location\_x = x;

m\_Location\_y = y;

}

---

## Member Function Documentation

|  |  |  |  |  |  |
| --- | --- | --- | --- | --- | --- |
| virtual void TAnimal::BeginStep | ( | void |  | ) |  |

inlinevirtual

Reimplemented from TALMaSSObject.

Reimplemented in Skylark\_Male, Skylark\_Female, Skylark\_PreFledgeling, Skylark\_Nestling, and Skylark\_Clutch.

{

}

|  |  |  |  |  |  |
| --- | --- | --- | --- | --- | --- |
| void TAnimal::CheckManagement | ( | void |  | ) |  |

Checks to see if there has been a management event at the TAnimals' x,y location. If so calls an event handler to handle the management event.

References sleep\_all\_day.

Referenced by Skylark\_Clutch::BeginStep(), Skylark\_Nestling::BeginStep(), Skylark\_PreFledgeling::BeginStep(), Skylark\_Female::BeginStep(), and Skylark\_Male::BeginStep().

{

FarmToDo event;

int i = 0;

while ( ( event = ( FarmToDo )m\_OurLandscape->SupplyLastTreatment( m\_Location\_x, m\_Location\_y, & i ) ) != sleep\_all\_day ) {

if ( OnFarmEvent( event ) ) break;

}

}

|  |  |  |  |
| --- | --- | --- | --- |
| void TAnimal::CheckManagementXY | ( | int | *x*, |
|  |  | int | *y* |
|  | ) |  |  |

Checks to see if there has been a management event at the x,y location. If so calls an event handler to handle the management event.

References sleep\_all\_day.

{

FarmToDo event;

int i = 0;

while ( ( event = ( FarmToDo )m\_OurLandscape->SupplyLastTreatment( x, y, & i ) ) != sleep\_all\_day ) {

OnFarmEvent( event );

}

}

|  |  |  |  |  |
| --- | --- | --- | --- | --- |
| virtual void TAnimal::CopyMyself | ( |  | ) |  |

inlinevirtual

{

};

|  |  |  |  |  |  |
| --- | --- | --- | --- | --- | --- |
| void TAnimal::CorrectWrapRound | ( | void |  | ) |  |

inlineprotected

Corrects wrap around co-ordinate problems.

Does the standard wrap around testing of positions. Uses the addition and modulus operators to avoid testing for negative or > landscape extent. This would be an alternative that should be tested for speed at some point.

References m\_Location\_x, m\_Location\_y, m\_OurLandscape, Landscape::SupplySimAreaHeight(), and Landscape::SupplySimAreaWidth().

{

m\_Location\_x = (m\_Location\_x + m\_OurLandscape->SupplySimAreaWidth()) % m\_OurLandscape->SupplySimAreaWidth();

m\_Location\_y = (m\_Location\_y + m\_OurLandscape->SupplySimAreaHeight()) % m\_OurLandscape->SupplySimAreaHeight();

}

|  |  |  |  |  |
| --- | --- | --- | --- | --- |
| virtual void TAnimal::Dying | ( |  | ) |  |

inlinevirtual

References KillThis().

Referenced by Skylark\_Population\_Manager::Catastrophe().

{

KillThis();

}

|  |  |  |  |  |  |
| --- | --- | --- | --- | --- | --- |
| virtual void TAnimal::EndStep | ( | void |  | ) |  |

inlinevirtual

Reimplemented from TALMaSSObject.

Reimplemented in Skylark\_Male, Skylark\_Female, Skylark\_PreFledgeling, Skylark\_Nestling, and Skylark\_Clutch.

{

}

|  |  |  |  |  |
| --- | --- | --- | --- | --- |
| virtual void TAnimal::KillThis | ( |  | ) |  |

inlinevirtual

References TALMaSSObject::CurrentStateNo, and TALMaSSObject::StepDone.

Referenced by Dying().

{

CurrentStateNo = -1; StepDone = true;

};

|  |  |  |  |  |  |
| --- | --- | --- | --- | --- | --- |
| virtual bool TAnimal::OnFarmEvent | ( | FarmToDo |  | ) |  |

inlinevirtual

Reimplemented in Skylark\_Male, Skylark\_Female, Skylark\_PreFledgeling, Skylark\_Nestling, and Skylark\_Clutch.

{

return false;

}

|  |  |  |  |  |  |
| --- | --- | --- | --- | --- | --- |
| void TAnimal::SetX | ( | int | *a\_x* | ) |  |

inline

References m\_Location\_x.

{

m\_Location\_x = a\_x;

}

|  |  |  |  |  |  |
| --- | --- | --- | --- | --- | --- |
| void TAnimal::SetY | ( | int | *a\_y* | ) |  |

inline

References m\_Location\_y.

{

m\_Location\_y = a\_y;

}

|  |  |  |  |  |  |
| --- | --- | --- | --- | --- | --- |
| virtual void TAnimal::Step | ( | void |  | ) |  |

inlinevirtual

Reimplemented from TALMaSSObject.

Reimplemented in Skylark\_Male, Skylark\_Female, Skylark\_PreFledgeling, Skylark\_Nestling, and Skylark\_Clutch.

{

}

|  |  |  |  |  |
| --- | --- | --- | --- | --- |
| int TAnimal::Supply\_m\_Location\_x | ( |  | ) |  |

inline

References m\_Location\_x.

Referenced by Skylark\_Population\_Manager::BreedingPairsOutput(), Population\_Manager::FindClosest(), CompareX::operator()(), Skylark\_Population\_Manager::TheCIPEGridOutputProbe(), Skylark\_Population\_Manager::TheNWordOutputProbe(), and Skylark\_Population\_Manager::TheRipleysOutputProbe().

{

return m\_Location\_x;

}

|  |  |  |  |  |
| --- | --- | --- | --- | --- |
| int TAnimal::Supply\_m\_Location\_y | ( |  | ) |  |

inline

References m\_Location\_y.

Referenced by Skylark\_Population\_Manager::BreedingPairsOutput(), CompareY::operator()(), Skylark\_Population\_Manager::TheCIPEGridOutputProbe(), Skylark\_Population\_Manager::TheNWordOutputProbe(), and Skylark\_Population\_Manager::TheRipleysOutputProbe().

{

return m\_Location\_y;

}

|  |  |  |  |  |
| --- | --- | --- | --- | --- |
| unsigned TAnimal::SupplyFarmOwnerRef | ( |  | ) |  |

Provides the farmer reference for the location of a TAnimal

{

return m\_OurLandscape->SupplyFarmOwner( m\_Location\_x, m\_Location\_y );

}

|  |  |  |  |  |
| --- | --- | --- | --- | --- |
| int TAnimal::SupplyPolygonRef | ( |  | ) |  |

inline

References m\_Location\_x, m\_Location\_y, m\_OurLandscape, and Landscape::SupplyPolyRef().

{

return m\_OurLandscape->SupplyPolyRef( m\_Location\_x, m\_Location\_y );

}

|  |  |  |  |  |
| --- | --- | --- | --- | --- |
| AnimalPosition TAnimal::SupplyPosition | ( |  | ) |  |

Provides the location of an animal in terms of x,y,elementtype and vegetation type

References AnimalPosition::m\_EleType, AnimalPosition::m\_VegType, AnimalPosition::m\_x, and AnimalPosition::m\_y.

{

AnimalPosition SkP;

SkP.m\_x = m\_Location\_x;

SkP.m\_y = m\_Location\_y;

SkP.m\_EleType = m\_OurLandscape->SupplyElementType( m\_Location\_x, m\_Location\_y );

SkP.m\_VegType = m\_OurLandscape->SupplyVegType( m\_Location\_x, m\_Location\_y );

return SkP;

}

|  |  |  |  |  |
| --- | --- | --- | --- | --- |
| virtual int TAnimal::WhatState | ( |  | ) |  |

inlinevirtual

Reimplemented in Skylark\_Base.

Referenced by CompareState::operator()().

{

return 0;

}

---

## Member Data Documentation

int TAnimal::m\_Location\_x

protected

Referenced by CorrectWrapRound(), Skylark\_Male::EstablishingATerritory(), Skylark\_Female::EstablishTerritory(), Skylark\_Base::InSquare(), Skylark\_Clutch::OnFarmEvent(), Skylark\_Nestling::OnFarmEvent(), Skylark\_Male::OnNestLocation(), SetX(), Skylark\_Female::st\_Arriving(), Skylark\_Female::st\_Finding\_Territory(), Skylark\_Male::st\_FindingTerritory(), Skylark\_Female::st\_Floating(), Skylark\_Male::st\_Floating(), Skylark\_Female::st\_Flocking(), Skylark\_Male::st\_Flocking(), Skylark\_Clutch::st\_Hatching(), Skylark\_Female::st\_Laying(), Skylark\_Female::st\_MakingNest(), Skylark\_Nestling::st\_Maturing(), Skylark\_PreFledgeling::st\_Maturing(), Skylark\_Female::Step(), Skylark\_Male::Step(), Supply\_m\_Location\_x(), and SupplyPolygonRef().

int TAnimal::m\_Location\_y

protected

Referenced by CorrectWrapRound(), Skylark\_Male::EstablishingATerritory(), Skylark\_Female::EstablishTerritory(), Skylark\_Base::InSquare(), Skylark\_Clutch::OnFarmEvent(), Skylark\_Nestling::OnFarmEvent(), Skylark\_Male::OnNestLocation(), SetY(), Skylark\_Female::st\_Arriving(), Skylark\_Female::st\_Finding\_Territory(), Skylark\_Male::st\_FindingTerritory(), Skylark\_Female::st\_Floating(), Skylark\_Male::st\_Floating(), Skylark\_Female::st\_Flocking(), Skylark\_Male::st\_Flocking(), Skylark\_Clutch::st\_Hatching(), Skylark\_Female::st\_Laying(), Skylark\_Female::st\_MakingNest(), Skylark\_Nestling::st\_Maturing(), Skylark\_PreFledgeling::st\_Maturing(), Skylark\_Female::Step(), Skylark\_Male::Step(), Supply\_m\_Location\_y(), and SupplyPolygonRef().

Landscape\* TAnimal::m\_OurLandscape

protected

Referenced by CorrectWrapRound(), Skylark\_Female::FeedYoung(), Skylark\_Male::GetFood(), Skylark\_Adult::GetVegHindrance(), Skylark\_Adult::GetWeatherHindrance(), Skylark\_Nestling::OnDeserted(), Skylark\_Clutch::OnFarmEvent(), Skylark\_Nestling::OnFarmEvent(), Skylark\_PreFledgeling::OnFarmEvent(), Skylark\_Female::OnFarmEvent(), Skylark\_Male::OnFarmEvent(), Skylark\_Female::OnMateDying(), Skylark\_Male::OnMateDying(), Skylark\_Female::OnMateHomeless(), Skylark\_Male::OnMateLeaving(), Skylark\_Nestling::OnYouHaveBeenEaten(), Skylark\_Female::st\_Arriving(), Skylark\_Male::st\_AttractingAMate(), Skylark\_Female::st\_BuildingUpResources(), Skylark\_Female::st\_CaringForYoung(), Skylark\_Male::st\_CaringForYoung(), Skylark\_Clutch::st\_Developing(), Skylark\_Nestling::st\_Developing(), Skylark\_Female::st\_Dying(), Skylark\_Female::st\_Emigrating(), Skylark\_Male::st\_Emigrating(), Skylark\_Female::st\_Floating(), Skylark\_Male::st\_Floating(), Skylark\_Female::st\_Flocking(), Skylark\_Male::st\_Flocking(), Skylark\_Female::st\_GivingUpTerritory(), Skylark\_Clutch::st\_Hatching(), Skylark\_Female::st\_Incubating(), Skylark\_Female::st\_Laying(), Skylark\_Female::st\_MakingNest(), Skylark\_Nestling::st\_Maturing(), Skylark\_PreFledgeling::st\_Maturing(), Skylark\_Female::st\_PreparingForBreeding(), Skylark\_Female::st\_StartingNewBrood(), Skylark\_Female::st\_StoppingBreeding(), Skylark\_Female::Step(), and SupplyPolygonRef().

---

The documentation for this class was generated from the following files:

- populationmanager.h
- PopulationManager.cpp


- TAnimal
- Generated on Thu Jan 10 2013 13:15:38 for ALMaSS Skylark ODdox by
   1.8.1.1
